# In Vitro Shear Bond Strength of Additively Manufactured Denture Base Resins to Hard Chairside Reline Materials

**DOI:** 10.1016/j.identj.2026.109568

**Published:** 2026-04-28

**Authors:** Je-Hyeon Yoo, Jimin Kim, Yeseul Park, Jee-Hwan Kim

**Affiliations:** a Department of Prosthodontics, Yonsei University Wonju Severance Christian Hospital, Wonju, Republic of Korea; b Department of Prosthodontics, Oral and Science Research Center, Yonsei University College of Dentistry, Seoul, Republic of Korea; c Department of Prosthodontics, Yonsei University College of Dentistry, Seoul, Republic of Korea

**Keywords:** Denture bases, Denture relining, Additive manufacturing, Shear bond strength, Thermocycling

## Abstract

**Introduction and aims:**

Reliable bonding between additively manufactured denture base resins and hard chairside reline materials is essential for long-term clinical performance, yet evidence under simulated aging conditions remains limited. This *in vitro* study evaluated the shear bond strength (SBS) of two hard chairside reline materials to conventional polymethyl methacrylate (PMMA) and two additively manufactured denture base resins under thermocycling conditions simulating relining at denture delivery and after clinical service.

**Methods:**

One heat-polymerized PMMA resin and two additively manufactured denture base resins composed of urethane dimethacrylate (UDMA) and methacrylate ester monomer (MA) were bonded to Tokuyama Rebase II (R) or Ufi Gel Hard (U), forming six material combinations. Specimens were assigned to three conditions: non-thermocycling (NT), thermocycling before relining (TB), or thermocycling after relining (TA). Ten specimens were prepared per subgroup (*n* = 180). Specimens that exhibited complete debonding during thermocycling were assigned an SBS value of 0 MPa and included in the analysis. SBS was measured using a universal testing machine, and failure modes were classified. Data were analysed using appropriate statistical tests (α = 0.05).

**Results:**

SBS was significantly influenced by denture base resin type, reline material, and thermocycling condition (*P* < .05). U demonstrated higher SBS than R across all denture base resins. Under NT conditions, the MA–U group showed the highest SBS (6.78 ± 1.57 MPa), and the UDMA–R group showed the lowest (1.41 ± 0.49 MPa). After thermocycling, 80% of UDMA–R and 20% of MA–R specimens debonded, whereas no failures occurred in the U groups. R predominantly exhibited adhesive failures, while U mainly showed cohesive or mixed failures.

**Conclusion:**

Within the limitations of this *in vitro* study, bonding performance between denture base resins and hard chairside reline materials was influenced by material compatibility and thermal aging. U demonstrated greater resistance to thermally induced debonding than R.

**Clinical relevance:**

Appropriate selection of hard chairside reline materials for additively manufactured dentures can provide bonding performance comparable to conventional PMMA.

## Introduction

Advances in computer-aided design (CAD) and computer-aided manufacturing (CAM) technologies have transformed prosthodontics, enabling the clinical application of additive manufacturing in removable prosthesis fabrication.^1^, ^2^, ^3^ Compared with subtractive milling, additive manufacturing allows efficient fabrication of patient-specific removable dentures while reducing material waste and production costs. However, evidence regarding the long-term clinical performance of additively manufactured dentures in relining procedures remains limited.^1^^,^^2^

Removable dentures play a critical role in oral rehabilitation, particularly for elderly or medically compromised patients who are ineligible for implant therapy.^4^ Although conventional heat-processed denture fabrication is technique-sensitive and labour-intensive, CAD/CAM-based digital workflows, including subtractive and additive manufacturing, have been introduced to improve standardization, reproducibility, and workflow efficiency.^5^ Nevertheless, additively manufactured dentures remain at a relatively early stage of clinical adoption. Therefore, their mechanical reliability, biological compatibility, and relining performance warrant further experimental validation.^6^

Denture relining is an essential clinical procedure used to compensate for alveolar bone resorption and mucosal adaptation, which can lead to loss of retention and occlusal instability. Depending on the clinical situation, relining is performed using soft or hard materials for restoring denture fit and function. Among these, durable bonding between hard chairside reline materials and denture bases is particularly critical for maintaining long-term clinical stability.^3^ This issue is particularly relevant for additively manufactured denture resins, as clinicians have expressed increasing concern regarding their potentially inferior bonding performance compared with conventional polymethyl methacrylate (PMMA).^7^ Therefore, a systematic evaluation of denture base and reline material combinations is required to establish reliable bonding protocols.

Clinically, chairside relining is performed in two scenarios: (1) on the day of denture delivery and (2) after a certain period of use. Evaluating shear bond strength (SBS) under both conditions is clinically important, as it may provide insight into the durability and stability of the bonded interface under simulated aging conditions. Beyond conventional bond strength measurements obtained after mechanical testing, early debonding events occurring during aging procedures may also provide clinically relevant information regarding interfacial durability.

Accordingly, this study aimed to evaluate the SBS of conventional PMMA and two additively manufactured denture base resins bonded to two hard chairside reline materials under thermocycling conditions simulating relining at denture delivery and after clinical use. The null hypotheses were that denture base resin type, chairside reline material, and thermocycling sequence would not influence SBS.

## Material and methods

### Study design and group classification

**Fig. 1:**
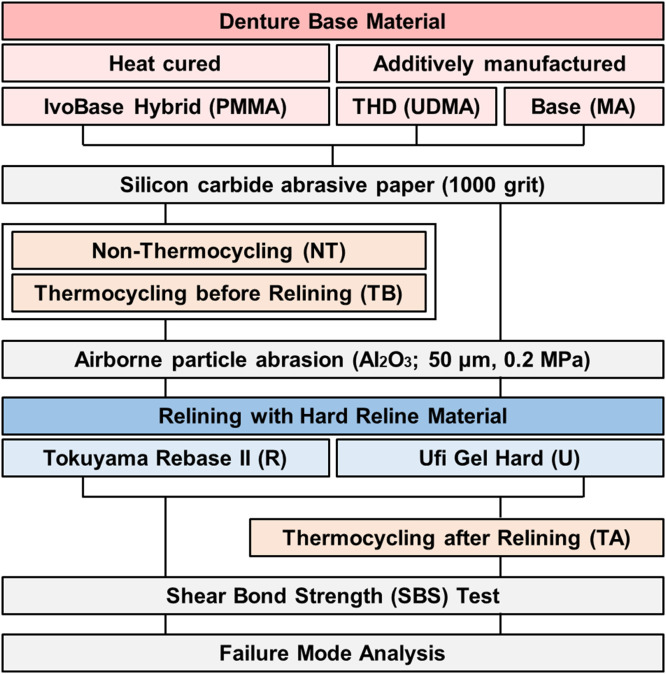
Workflow of the experimental protocol. PMMA, polymethyl methacrylate; UDMA, urethane-dimethacrylate; MA, methacrylate ester monomer; NT, non-thermocycling; TB, thermocycling before relining; TA, thermocycling after relining.

The group classification and experimental workflow are illustrated in Figure 1. Three denture base resins – one conventional heat-processed base resin and two additively manufactured resins – were paired with two hard chairside reline materials, yielding six groups for shear bond strength (SBS) testing. The material properties and group designations are summarized in Table 1. The three denture bases used PMMA, urethane-dimethacrylate (UDMA), and methacrylate ester monomer (MA), based on the main composition of the material. The two reline materials used were Tokuyama Rebase II (R) and Ufi Gel Hard (U). Specimens were assigned to three conditions: non-thermocycling (NT), thermocycling before relining (TB), and thermocycling after relining (TA). The NT group was not thermocycled. In the TB group, thermocycling was performed prior to relining, whereas in the TA group, relining was performed first followed by thermocycling. All specimens were then subjected to SBS testing. These groups were labelled X–Y–Z, where X denotes the thermocycling condition, Y the denture base resin, and Z the reline material.

**Table 1:** Materials, compositions, and manufacturers of denture base resins and hard chairside reline materials.

Group	Material	Composition		Manufacturer
PMMA	IvoBase Hybrid	Powder	PMMA, Softener (non-phthalate), Initiator, Pigments	Ivoclar, Schaan, Liechtenstein
		Liquid	MMA, Dimethacrylate (cross-linking agent), Catalyst	

UDMA	Tera Harz Denture Base	UDMA, Urethane acrylate oligomer		Graphy, Seoul, Korea

MA	Base	Methacrylate ester monomer, (Octahydro-4,7-methano-1H-indenediyl)bis(methylene)diacry late, 3,3,5-trimethylcyclohexyl		NextDent, Soesterberg, Netherlands

R	Tokuyama Rebase II (Normal)	Powder	PEMA	Tokuyama Dental, Tokyo, Japan
		Liquid	AAEMA, 1,9-NDMA	
		Adhesive	Ethyl-acetate, Acetone	

U	Ufi Gel Hard	Powder	PEMA	Voco Dental, Cuxhaven, Germany
		Liquid	1,6-HDMA	
		Adhesive	Acetone, 2-HEMA	

1,6-HDMA, 1,6-hexanediol dimethacrylate; 1,9-NDMA, 1,9-nonanediol dimethacrylate; 2-HEMA, 2-hydroxyethyl methacrylate; AAEMA, 2-(acetoacetoxy)ethyl methacrylate; MMA, methyl methacrylate; PEMA, polyethyl methacrylate; PMMA, polymethyl methacrylate; UDMA, urethane-dimethacrylate.

### Sample preparations

1) *Specimen design:* All specimens were prepared using CAD software (TinkerCAD; Autodesk Inc.). No ISO or other international standard specifies specimen dimensions for SBS testing. The denture base specimens were designed as rectangular solids measuring 10 × 10 × 6 mm based on dimensions commonly adopted in previous in vitro studies.^8^ For SBS testing, a cylindrical mold (21 × 10 mm) was designed to fit the testing jig in accordance with the manufacturer’s instructions. A slot was incorporated at the centre of the mould to accommodate the denture base specimen, ensuring consistent bonding location and load application across all specimens.
2) *Specimen grouping and sample size:* A total of 60 specimens were fabricated for each denture base resin (*n* = 10 per subgroup). The sample size was determined based on previously published *in vitro* studies with comparable experimental designs and outcome measures.^8^, ^9^, ^10^ The specimens were prepared according to the manufacturer’s instructions.
3) Fabrication of denture base specimens
 - *Heat-polymerized PMMA specimens:* For PMMA specimens, a rectangular wax block (55 × 45 × 13 mm) was positioned in the flask containing dental stone. The mixed resin was injected using a pressure system (IvoBase Injector; Ivoclar Vivadent) and polymerized for 35 min at 0.6 MPa. The specimens were then trimmed to 10 × 10 × 6 mm using precision milling.
 - *Two additively manufactured denture base specimens:* For additively manufactured materials, denture base specimens were fabricated from Standard Tessellation Language (STL) files using two different vat-polymerization–based manufacturing systems. After fabrication, all specimens were washed with 95% isopropyl alcohol for 5 min in an ultrasonic cleaner (Twin Tornado; Medifive) and post-polymerized using manufacturer-recommended light-curing units and protocols. All post-processing procedures, including washing and post-polymerization, were performed strictly in accordance with the manufacturers’ recommended guidelines for each denture base resin, without any modification. For both additively manufactured specimens, the relining surface was positioned horizontally on the build platform, with support structures oriented vertically, perpendicular to the occlusal surface, to ensure a clearly defined and standardized bonding surface.
  a) *Digital Light Processing (DLP)-fabricated MA specimens*: For the MA group, STL files were imported into CAD software (3D Sprint v2.13; NextDent) and printed using a DLP-based manufacturing device (NextDent 5100; NextDent). The layer thickness was set to 100 µm, with an XY resolution of 65 µm and a wavelength of 405 nm. Post-polymerization was performed for 30 min using a light-curing unit (LC-3DPrint Box; NextDent).
  b) *Liquid Crystal Display (LCD)-fabricated UDMA specimens*: For the UDMA group, STL files were imported into printing software (UNIZ Maker; UniZ Technology) and specimens were fabricated using an LCD-based manufacturing device (Uniz Nbee; UniZ Technology) with a UDMA-based denture base resin (S-100M; Graphy). The layer thickness was set to 100 µm, with an XY resolution of 49.8 µm and a wavelength of 405 nm. Post-polymerization was performed for 20 min using a post-polymerization unit (Cure-M 102H; Sona Global Co., Ltd).

### Specimen embedding and surface treatment

Embedding moulds were fabricated using a UDMA-based resin to ensure dimensional stability during polymerization and thermocycling. Each denture base specimen was centrally embedded in a cylindrical mold using an autopolymerizing acrylic resin (Ortho-jet; Lang Dental Manufacturing Co.), leaving the bonding surface exposed. Specimens were positioned such that the bonded interface was oriented parallel to the loading direction, allowing standardized shear force application during testing. Specimen alignment was visually confirmed prior to polymerization to ensure consistent positioning across all groups. To standardize the bonding surface, the exposed denture base surface was ground under running tap water using 1000-grit silicon carbide paper at 300 rpm and subsequently ultrasonically cleaned for 3 min (SD-120H; Sungdong Ultrasonic Co.). Mechanical surface treatment was performed by airborne-particle abrasion (APA) using a sandblasting unit (SJPB00; Sejong Dental) with 50 µm aluminum oxide particles applied perpendicularly at a distance of 10 mm, under 0.2 MPa for 10 s. After air abrasion, bonding surfaces were ultrasonically cleaned for 3 min and air-dried before application of the reline material.

### Application of hard chairside reline materials

Hard chairside reline materials were mixed according to the manufacturer’s instructions. The proprietary adhesive supplied with each hard chairside reline material was used according to the manufacturer’s instructions, and the composition and manufacturer of each adhesive are summarized in Table 1. The adhesive was applied to the specimen surface, after which the hard reline material was placed into a 6 mm diameter gelatin capsule (150 mg) and positioned at the centre of the surface. Excess materials beyond the capsule margins were removed before polymerization. After the working time had elapsed, the specimens were immersed in warm water for 3 min to complete polymerization and dissolve the gelatin capsule. For R, the resin hardener was added to the warm water during immersion after relining, in accordance with the manufacturer’s instructions. For U, the same immersion procedure was performed without the addition of any hardener.

### Thermocycling

Specimens underwent thermocycling either before relining (TB) or after relining (TA) with the reline material. To simulate approximately 6 months of clinical use,^11^^,^^12^ thermocycling was performed according to group assignment. Thermocycling consisted of 5000 cycles alternating between 5 °C and 55 °C water baths, with a 30-s dwell time and a 5-s transfer interval. NT specimens were stored in distilled water at 37 °C for 24 h after relining.

### Shear bond strength (SBS) test

**Fig. 2:**
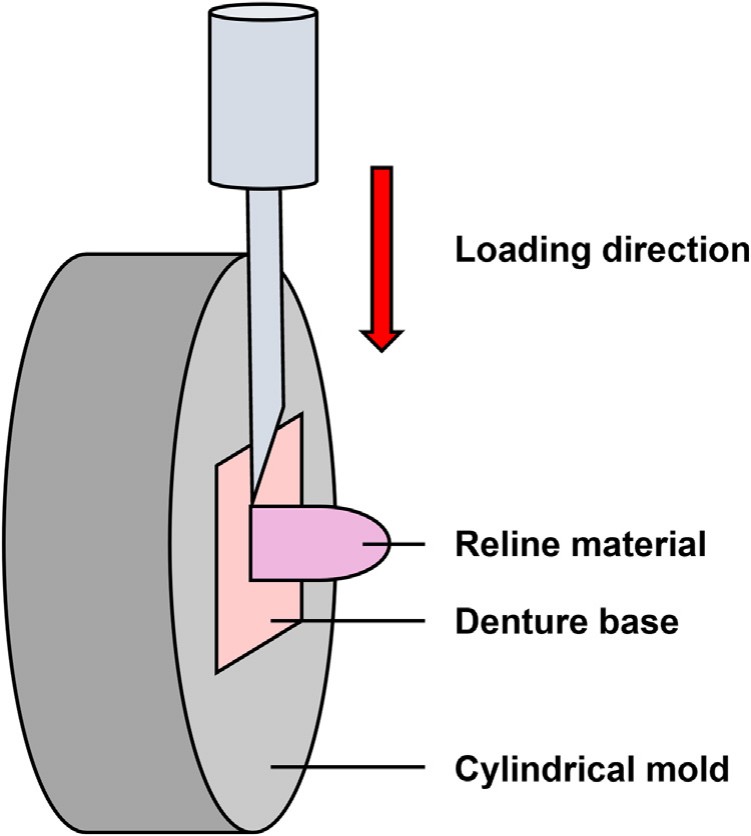
Schematic illustration of the shear bond strength test setup. Specimens were positioned such that the bonded interface at the reline–denture base junction was oriented perpendicular to the loading direction, allowing shear stress generation at the bonded interface. Load was applied using a universal testing machine at a crosshead speed of 1 mm/min to generate shear stress at the bonded interface.

A schematic illustration of the SBS testing configuration, including the direction of load application relative to the bonded interface, is provided in Figure 2. A universal testing machine (EZ-LX HS; Shimadzu Corp.) applied load at a crosshead speed of 1 mm/min until the occurrence of bond failure. No ISO or other international standard specifies specimen dimensions for SBS testing of chairside hard reline materials bonded to denture base resins. Accordingly, the specimen geometry used in this study was based on dimensions commonly adopted in previous *in vitro* studies. SBS was calculated using the following formula:

$$SBS\;\left(MPa\right)=F/A\;\left[*F=\text{maximum}\;\text{load}\;\left(N\right)\;/\;A=\text{bonded}\;\text{area}\;\left(28.26\;mm^{2}\right)\right]$$

The bonded area was defined by the standardized mould geometry. Minor deviations at the bonded interface after specimen preparation and thermocycling were not quantified. Specimens that exhibited complete debonding during thermocycling were classified as pre-test failures. These specimens were assigned an SBS value of 0 MPa and included in the statistical analysis to represent complete bond failure under thermal aging conditions.

### Failure mode analysis

Following SBS testing, failure modes were initially classified based on visual inspection. Adhesive failure was defined as separation at the interface between the denture base resin and the reline material. Cohesive failure was defined as fracture confined within either the denture base resin or the reline material. Mixed failure was defined as fracture of the denture base resin or reline material accompanied by interfacial separation. The failure surfaces were further examined and photographed using an optical microscope at 10 × magnification (SMZ161; Motic) equipped with image acquisition software (Motic Images Plus 3.0; Motic). Microscopic examination was used to confirm the visually assigned failure modes. Failure mode analysis was performed for descriptive purposes only and was not subjected to statistical comparison.

### Statistical analysis

All statistical analyses were performed using IBM SPSS Statistics (version 26.0; IBM Corp.). Data normality was assessed using the Shapiro–Wilk test, and homogeneity of variances was evaluated using Levene’s test prior to analysis of variance (ANOVA). Specimens that completely debonded during thermocycling were assigned an SBS value of 0 MPa and included in the statistical analysis. For the NT and TB conditions, which satisfied the normality assumption, two-way ANOVA was used to evaluate the main effects of denture base resin and reline material and their interaction under identical thermocycling conditions. For the TA condition, where deviation from normality was observed after inclusion of pre-test failures, non-parametric analyses were applied. Differences among denture base resins were evaluated using the Kruskal–Wallis test followed by pairwise comparisons with Bonferroni adjustment, and comparisons between reline materials were performed using the Mann–Whitney U test. The significance level was set at α = 0.05.

## Results

Two-way ANOVA results for the NT and TB conditions are presented in Tables 2 and 3. Under NT conditions, significant main effects of denture base resin type (F = 37.954, *P* < .001) and reline material (F = 42.489, *P* < .001) on SBS were observed. A significant interaction between denture base resin and reline material was also detected (F = 5.144, *P* = .009) (Table 2). Under TB conditions, the main effect of reline material remained significant (F = 71.058, *P* < .001), whereas the main effect of denture base resin was not significant (F = 0.505, *P* = .606). No significant interaction effect between denture base resin and reline material was observed (F = 2.045, *P* = .139) (Table 3).

**Table 2:** Results of two-way ANOVA in non-thermocycling (NT) condition.

	Sum of squares	Df	Mean squares	F	*P*-value
Denture base materials	92.557	2	46.289	37.954	.000
Reline materials	51.820	1	51.820	42.489	.000
Denture base materials × Reline materials	12.546	2	6.273	5.144	.009
Error	65.858	54	1.220	-	-

**Table 3:** Results of two-way ANOVA in the thermocycling before relining (TB) condition.

	Sum of squares	Df	Mean squares	F	*P*-value
Denture base materials	2.144	2	1.072	0.505	.606
Reline materials	150.765	1	150.765	71.058	.000
Denture base materials × Reline materials	8.678	2	4.339	2.045	.139
Error	114.572	54	2.122	-	-

The SBS values and pre-test failure rates are summarized in Table 4. Under NT conditions, significant differences in SBS were observed among denture base resins within both R and U groups (*P* < .05). In the R groups, MA–R exhibited significantly higher SBS than PMMA–R and UDMA–R, whereas UDMA–R showed the lowest bond strength. In the U groups, MA–U demonstrated significantly higher SBS than PMMA–U and UDMA–U. When reline materials were compared within the same denture base resin, U showed significantly higher SBS than R for UDMA and MA (*P* < .05), whereas no significant difference was detected between PMMA–R and PMMA–U. No pre-test failures occurred in any NT subgroup.

**Table 4:** Mean ± standard deviation (SD) of shear bond strength (MPa) between denture base resins and hard chairside reline materials under different thermocycling conditions.

		PMMA	UDMA	MA
NT	R	4.00 ± 1.23 B,a	1.41 ± 0.49 C,b	5.24 ± 1.04 A,b
	U	4.93 ± 0.63 B,a	4.52 ± 1.27 B,a	6.78 ± 1.57 A,a

TB	R	2.37 ± 0.79 A,b	1.45 ± 0.48 A,b	1.21 ± 0.56 A,b
	U	4.62 ± 1.90 A,a	4.61 ± 2.48 A,a	5.32 ± 1.35 A,a

TA	R	1.98 ± 0.95 A,b	0.07 ± 0.16 B,b (20% remaining)	0.55 ± 0.34 B,b (80% remaining)
	U	5.21 ± 0.57 A,a	3.86 ± 0.79 B,a	4.85 ± 0.73 A,a

Specimens that exhibited complete debonding during thermocycling were assigned an SBS value of 0 MPa and included in the statistical analysis. Different uppercase letters indicate statistically significant differences among denture base resins within the same reline material and thermocycling condition, whereas different lowercase letters indicate statistically significant differences between reline materials within the same denture base resin and thermocycling condition. Statistical comparisons were performed separately for each thermocycling condition (NT, TB, and TA). Values in parentheses indicate the proportion of specimens remaining for SBS measurement after thermocycling. PMMA, polymethyl methacrylate; UDMA, urethane-dimethacrylate; MA, methacrylate ester monomer; U, Ufi Gel Hard; R, Tokuyama Rebase II; NT, non-thermocycling; TB, thermocycling before relining; TA, thermocycling after relining.

Under TB conditions, no significant differences in SBS were observed among denture base resins within either reline material group (*P* > .05). However, for each denture base resin, U showed significantly higher SBS than R (*P* < .05). No complete debonding occurred in TB subgroups. Under TA conditions, SBS decreased markedly in R-relined groups after thermocycling. In the TA–UDMA–R subgroup, 80% of specimens, and in TA–MA–R, 20% of specimens exhibited complete debonding during thermocycling and were assigned an SBS value of 0 MPa. No pre-test failures occurred in U-relined groups under TA conditions. Within the R groups, SBS differed significantly among denture base resins (*P* < .001), with UDMA–R and MA–R showing lower SBS than PMMA–R. Within the U groups, significant differences were also observed among denture base resins (*P* < .001), with PMMA–U and MA–U demonstrating higher SBS than UDMA–U. Comparisons between reline materials under TA conditions showed significantly higher SBS for U than for R across all denture base resins (*P* < .001).

Thermocycling significantly reduced SBS in R-relined groups (*P* < .001), with the most pronounced reduction observed in the UDMA–R subgroup, in which the majority of specimens debonded. In contrast, SBS values in U-relined groups remained relatively stable after thermocycling, and a significant difference between NT and TA conditions was observed only for MA–U.

**Fig. 3:**
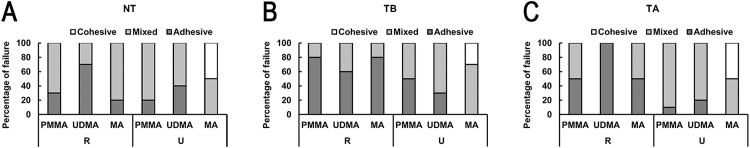
Distribution of the failure modes for all groups: A, denture base resins and reline materials under non-thermocycling. B, denture base resins and relining materials under thermocycling before relining. C, denture base resins and reline materials under thermocycling after relining. PMMA, polymethyl methacrylate; UDMA, urethane-dimethacrylate; MA, methacrylate ester monomer; U, Ufi Gel Hard; R, Tokuyama Rebase II; NT, non-thermocycling; TB, thermocycling before relining; TA, thermocycling after relining.

Failure mode analysis revealed predominantly adhesive failures in R-relined groups, whereas cohesive or mixed failures predominated in U-relined groups (Figure 3). Under TA conditions, complete adhesive separation occurred in 80% of UDMA–R specimens and 20% of MA–R specimens, whereas no complete debonding was observed in U-relined groups. Failure mode evaluation was descriptive in nature and was not subjected to inferential statistical analysis.

## Discussion

The results of this study demonstrated that SBS was significantly influenced by the type of denture base resin, the hard chairside reline material, and the thermocycling sequence, leading to rejection of the proposed null hypotheses. These findings indicate that bond strength depends on both material properties and compatibility.^13^^,^^14^ The two thermocycling configurations were designed to reflect distinct clinical relining scenarios. The TB condition simulates relining of dentures after a period of clinical service, whereas the TA condition represents relining performed at denture delivery, followed by thermocycling to simulate subsequent clinical service. Thermocycling influenced bonding behaviour differently depending on the timing of relining relative to simulated clinical service. This finding underscores the importance of material compatibility when interpreting *in vitro* bonding performance under aging conditions.^15^

The selection of denture base resins and hard chairside reline materials was based on frequent clinical application and practitioner preference.^16^ PMMA was used as the control, reflecting its long-standing clinical reliability.^17^ UDMA and MA-based additively manufactured denture base resins are promising materials owing to their favourable mechanical properties, printability, aesthetic versatility, and compliance with standardized performance requirements.^18^^,^^19^ Bonding performance varied with additively manufactured denture base resins.^20^ The densely cross-linked structure and low surface polarity of UDMA limit monomer penetration and chemical bonding with reline materials.^18^ MA exhibits limitations related to water sorption, dimensional changes, and progressive loss of mechanical stability.^21^ Although UDMA and MA exhibited distinct surface chemistries from PMMA, their bonding performance with hard chairside reline materials was comparable to PMMA under certain experimental conditions. This suggests that properly formulated additively manufactured resins may demonstrate bonding behaviour comparable to PMMA under standardized *in vitro* bonding protocols.^22^^,^^23^ Nevertheless, variations in the SBS across different reline materials highlight the importance of material compatibility for durable bonding with denture bases.^7^ Evidence on their bonding performance with hard chairside reline materials under simulated clinical conditions is limited, warranting further investigation.^7^^,^^24^^,^^25^

These mechanistic interpretations should be considered speculative rather than directly confirmed, as no chemical or interfacial analyses were performed. The superior bonding performance observed with U across different denture base resins may be attributed to its molecular composition and enhanced diffusion capability, whereas the bonding performance of R appears to depend on the substrate and testing conditions. Penetration of reline material into the denture base has been identified as a key determinant of bond strength.^26^ Both U and R are polyethyl methacrylate-based, methyl methacrylate-free formulations designed to reduce mucosal irritation.^27^ U contains low-molecular-weight hydrophilic components, including 2-hydroxyethyl methacrylate, 1,6-hexanediol dimethacrylate, and acetone. These components promote surface wetting and diffusion into the denture base, facilitating the formation of an interpenetrating polymer network (IPN).^27^^,^^28^ In contrast, the R liquid contains acetoacetoxyethyl methacrylate and 1,9-nonanediol dimethacrylate, along with ethyl acetate and acetone as adhesive components. The relatively higher molecular weight and lower mobility of these functional methacrylate and dimethacrylate monomers may limit their diffusion into the pre-polymerized denture base, resulting in less favourable IPN formation and reduced interfacial adaptation, particularly after aging.^29^^,^^30^ Thus, lower molecular weight monomers may facilitate greater penetration into denture bases, potentially improving bond strength through surface swelling and pore formation.^30^

Thermocycling induces repeated thermal expansion and contraction, generating interfacial stresses that may compromise bonding stability.^12^^,^^31^ Under thermally induced aging, the bonding performance of R appeared more susceptible to degradation, whereas U showed relatively less reduction in interfacial integrity under comparable conditions.^32^ The bonding stability observed with U is seemingly related to its adhesive composition, which facilitates surface wetting and diffusion into the denture base, rather than to differences in the cross-linking agent concentration.^33^ In contrast, the chemical composition of R may hinder intimate interfacial adaptation, which could contribute to stress accumulation and premature debonding.^34^ This vulnerability was more pronounced for the UDMA-based denture base resin, in which the rigidity of the highly cross-linked polymer network may have contributed to increased interfacial stress concentration during thermocycling.

The results suggest reduced compatibility between UDMA and R under the tested conditions. The highly cross-linked UDMA network may restrict chain mobility and limit methacrylate monomer diffusion, hindering the formation of IPNs essential for bonding.^35^ Although thermocycling can transiently enhance R infiltration through partial hydration, the low water sorption and reduced plasticization of UDMA favor concentration of thermal stress at the adhesive interface, contributing to bond degradation. Freshly processed UDMA substrates present an additional challenge because their relatively stable surfaces resist softening by solvents, which can result in early debonding.^36^ Taken together, these findings indicate that materials with broader chemical affinity, such as U, exhibit more consistent bonding behaviour across different denture base substrates under *in vitro* conditions.^37^

Failure patterns in bond testing are typically classified as adhesive, cohesive, or mixed.^38^ The effectiveness of bonding is influenced by multiple factors, including chemical composition, resin type, thermocycling, and surface treatment methods. These factors are fundamental considerations when interpreting the durability of denture base–reline material bonding under laboratory aging conditions.^39^ In the present study, cohesive failure predominated in the MA–U groups, reflecting strong adhesion, whereas adhesive failure dominated in the R groups, particularly after thermocycling. Adhesive separation under the TA condition resulted in pre-test failures in eight UDMA–R and two MA–R specimens, which were assigned an SBS value of 0 MPa and included in the analysis. These extensive early failures align with the observed incompatibility between specific denture base–reline material combinations and underscore the susceptibility of certain interfaces to thermally induced degradation.^10^ Bond strength and failure mode should therefore be considered together when interpreting the potential durability of relined denture systems under *in vitro* conditions, rather than as direct indicators of long-term clinical performance.^13^ By incorporating pre-test failures as 0 MPa rather than excluding them, the present study treats early debonding as a true adhesive failure and provides a more realistic representation of bond durability under thermocycling conditions. From an experimental standpoint, the high incidence of complete debonding itself may be more informative than the residual SBS values, emphasizing the limited durability of certain denture base–reline material combinations within the constraints of *in vitro* thermocycling.

The absence of standardized clinical SBS thresholds for hard reline materials makes interpretation of results challenging. Earlier benchmarks derived from soft-liner testing (∼0.45 MPa) are not applicable to hard relining bonding.^40^^,^^41^ Recent reports have reported SBS values in the range of approximately 10 to 16 MPa for PMMA under similar *in vitro* testing conditions. In contrast, additively manufactured denture base resins generally exhibit lower SBS values of approximately 5 MPa, which are often associated with cohesive or mixed failure modes, providing a useful comparative reference for *in vitro* testing.^24^^,^^32^^,^^42^ While the present results do not represent a definitive clinical threshold, they offer a practical experimental baseline for future laboratory investigations and underscore the need to establish standardized criteria for assessing bonding performance between hard reline materials and denture base resins.

This study has some limitations, including the evaluation of only two additively manufactured denture base resins with a limited sample size. The sample size of 10 specimens per subgroup was selected based on protocols commonly used in previous *in vitro* bond strength studies.^24^^,^^32^ However, no *a priori* power analysis was performed, and the relatively limited sample size should be considered when interpreting the findings. In addition, the macro-shear bond strength test configuration may induce non-uniform stress distribution at the bonded interface, which warrants cautious interpretation of absolute SBS values, particularly when comparing different material systems. The simplified specimen geometry used in this study did not replicate the complex morphology of actual dentures, which may limit direct extrapolation of the present findings to clinical conditions. Future studies should include larger sample sizes and a broader range of denture base resins to provide a more comprehensive understanding of bonding behaviour. The development of reline materials and adhesive systems specifically optimized for highly cross-linked additively manufactured denture base resins and exploration of pretreatment methods beyond APA are warranted.

## Conclusions

Within the limitations of this *in vitro* study, SBS between denture base resins and hard chairside reline materials was primarily influenced by material compatibility and thermocycling conditions. Certain additively manufactured denture base resin–reline material pairings demonstrated bonding performance comparable to conventional PMMA under specific experimental conditions. However, substantial variation was observed across reline materials and simulated aging conditions, and some material combinations showed pronounced reductions in bond stability after thermocycling. The inclusion of early debonding events as 0 MPa values further highlighted the susceptibility of specific interfaces to thermally induced degradation. These findings indicate that material compatibility, rather than denture base fabrication method alone, is a primary determinant of bonding durability. Accordingly, careful selection of compatible hard chairside reline materials is essential to achieve reliable bonding performance for both conventional and additively manufactured denture base resins.

## Author contributions

**Je-Hyeon Yoo:** Investigation, Project Administration, Software, Validation, Visualization, Writing – Original Draft. **Jimin Kim:** Data curation, Investigation, Software, Formal analysis, Validation, Writing – Original Draft. **Yeseul Park:** Data curation, Formal analysis, Visualization, Writing – Original Draft. **Jee-Hwan Kim:** Conceptualization, Funding acquisition, Methodology, Resources, Supervision, Writing – Review & Editing.

## Declaration of generative AI and AI-assisted technologies in the writing process

No generative AI tools were used in the preparation of this manuscript.

## Funding

This work was supported by the Yonsei University College of Dentistry Fund [grant number 6-2025-0014].

## Conflict of interest

None disclosed.

## Appendix Supplementary materials

Image, application 1
