# Supplementary material for: In Vitro Shear Bond Strength of Additively Manufactured Denture Base Resins to Hard Chairside Reline Materials
Source: Int Dent J. 2026 Apr 28;76(4):109568. doi: 10.1016/j.identj.2026.109568 (PMC13140052; doi:10.1016/j.identj.2026.109568)

**Supplementary Materials**

**Supplementary Figure 1**. Optical microscope images of representative specimens from each subgroup after shear bond strength testing on specimens relining with R. A, Adhesive failure modes of PMMA specimens. B, Mixed failure modes of PMMA specimens. C, Adhesive failure modes of UDMA specimens. D, Mixed failure modes of UDMA specimens. E, Adhesive failure modes of MA specimens. F, Mixed failure modes of MA specimens. PMMA, polymethyl methacrylate; UDMA, urethane-dimethacrylate; MA, methacrylate ester monomer; R, Tokuyama Rebase II.


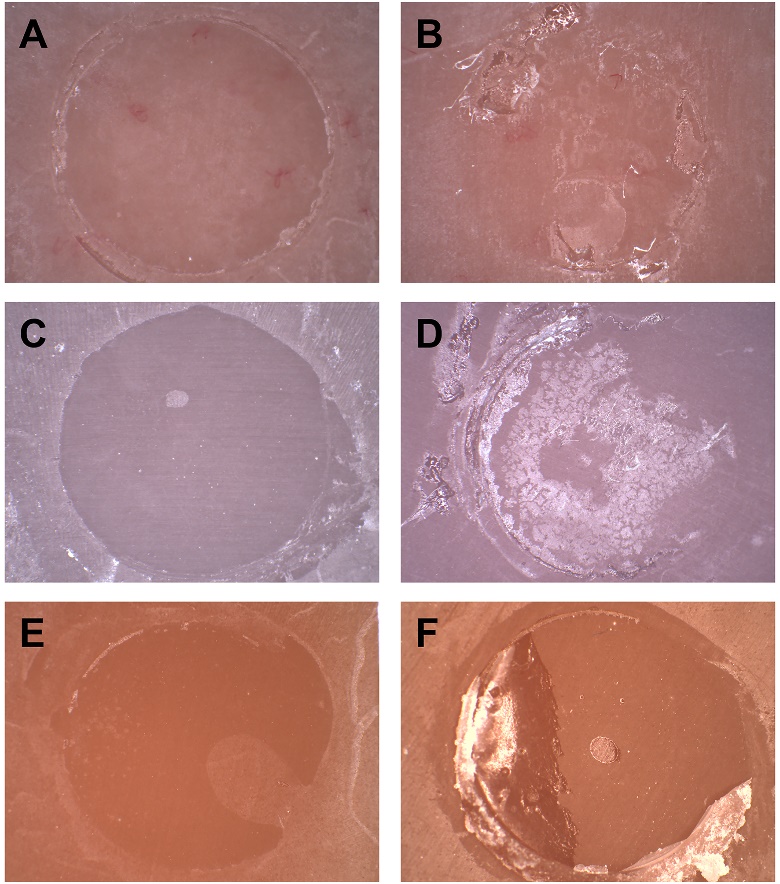


**Supplementary Figure 2.** Optical microscope images of representative specimens from each subgroup after shear bond strength testing on specimens relining with U. A, Adhesive failure modes of PMMA specimens. B, Mixed failure modes of PMMA specimens. C, Adhesive failure modes of UDMA specimens. D, Mixed failure modes of UDMA specimens. E, Cohesive failure modes of MA specimens. F, Mixed failure modes of MA specimens. PMMA, polymethyl methacrylate; UDMA, urethane-dimethacrylate; MA, methacrylate ester monomer; U, Ufi Gel Hard.


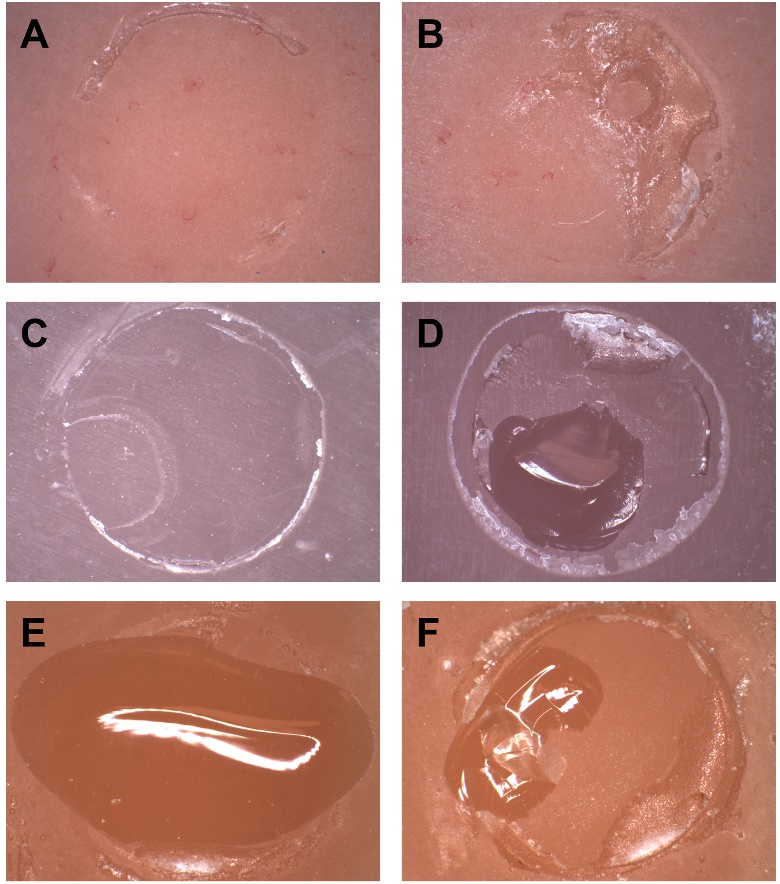

Supplement: Supplementary file 1 [file mmc1.docx]
